# Mesenteric Cyst in a 17-Month-Old Child: A Report of a Rare Case

**DOI:** 10.7759/cureus.106435

**Published:** 2026-04-04

**Authors:** Munir Malik, Eman Binte Arshad, Zainab Khan, Rabia Saleem

**Affiliations:** 1 Pediatrics, Shifa International Hospital Islamabad, Islamabad, PAK; 2 Medicine, Shifa Tameer-E-Millat University, Shifa College of Medicine, Islamabad, PAK; 3 Histopathology, Shifa International Hospital Islamabad, Islamabad, PAK

**Keywords:** abdominal distension, acute abdomen, hemorrhagic cyst, histopathology, mesenteric cyst, pediatric abdominal mass, pediatric surgery, surgical excision

## Abstract

Mesenteric cyst is a rare benign cystic lesion of childhood that has a range of presentations varying from asymptomatic to acute abdomen. Hence, it is often an incidental finding or missed in diagnosis. Very few cases with complications have been reported. The underlying etiology still remains unknown despite multiple theories. Definitive diagnosis requires histopathological confirmation. We present a case of a 17-month-old girl with a large symptomatic hemorrhagic cyst who underwent complete excision of the cyst.

## Introduction

Mesenteric cysts are benign intra-abdominal cystic lesions arising from the mesentery and are often considered part of a spectrum of lymphatic malformations. They are uncommon in the pediatric population and can occur anywhere along the mesentery of the gastrointestinal tract. Their presentation varies widely, ranging from incidental asymptomatic findings to acute abdomen [1]. In children, they are often overlooked as a differential diagnosis because their presentation mimics that of other more common abdominal pathologies, posing significant challenges in reaching an early and definitive diagnosis [2]. Surgery remains the mainstay of treatment as it decreases the risk of recurrence and complications [2]. We present a case of a mesenteric cyst in the subhepatic region of a 17-month-old girl.

## Case presentation

A 17-month-old female child presented to the outpatient department on 1 September 2025 with complaints of an intra-abdominal mass in the upper abdomen and abdominal pain for the past one week. The mass was initially localized to the midline and then moved to the right hypochondrium, with progressive increase in size. It was soft in consistency, mobile, and had regular borders. Pain was localized to the right hypochondrium, graded 10/10 on the pain intensity scale, and associated with a low-grade fever documented up to 99°F. It was reported that the child cried hysterically and withdrew even to a light touch. The frequency of stool passage had decreased to once every two days, and stool consistency was hard. Additionally, she had poor oral intake and generalized weakness. There was no history of nausea or vomiting.

**Figure 1:**
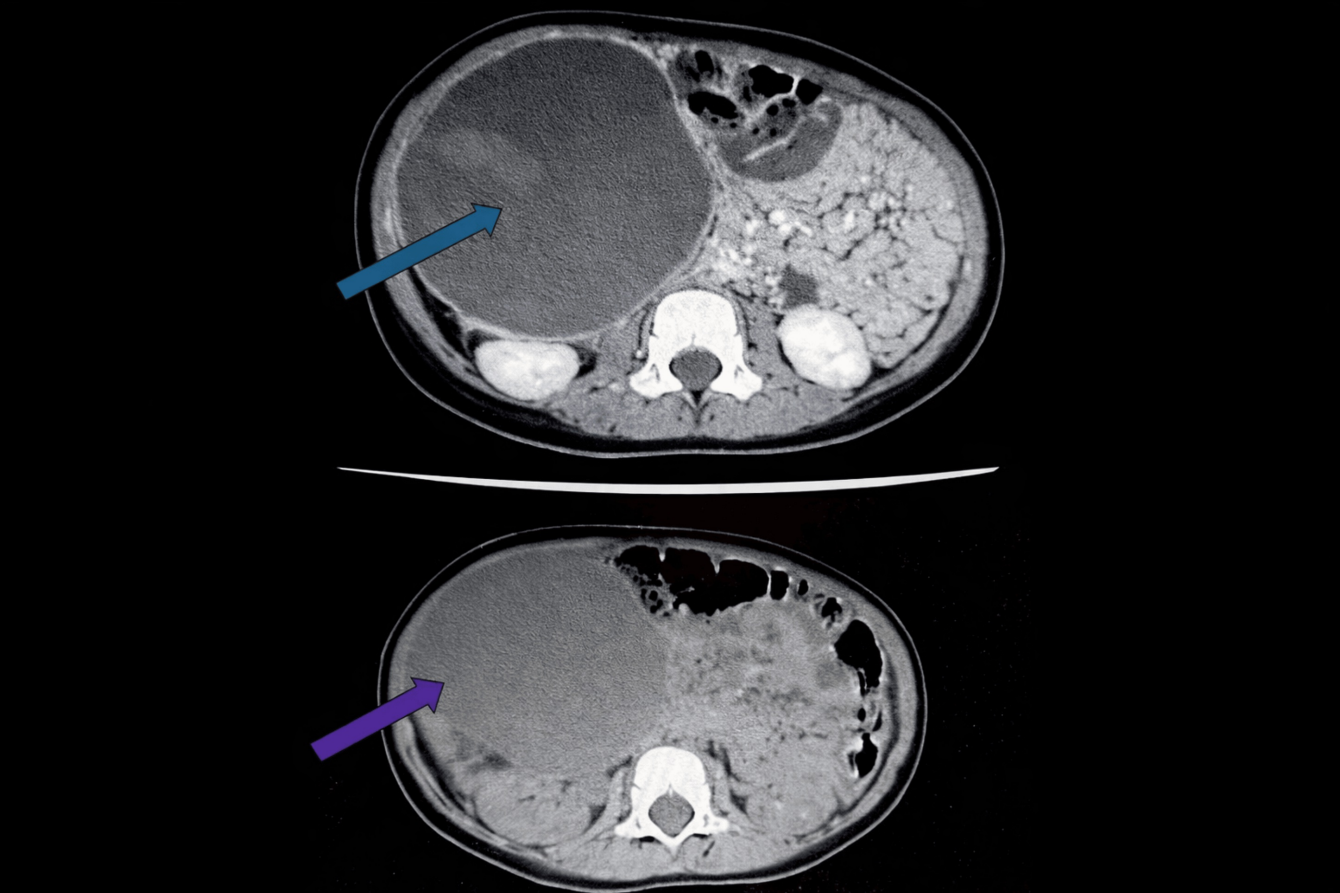
CT of the abdomen and pelvis with contrast (top, blue arrow), and without contrast (bottom, purple arrow) shows a large well-circumscribed hypodense cystic lesion in the right subhepatic region associated with mass effect on liver, gallbladder, bowel, and abdominal wall.

Ultrasound showed a large cystic lesion adjacent to the left hepatic lobe. CT of the abdomen and pelvis with and without contrast demonstrated a large, well-circumscribed cystic mass in the right subhepatic region, associated with compressive effects on the liver, gallbladder, bowel, and abdominal wall, raising suspicion for enteric duplication cyst, peritoneal mesothelial cyst, and cystic lymphangioma while ruling out ovarian causes (Figure 1).

**Figure 2:**
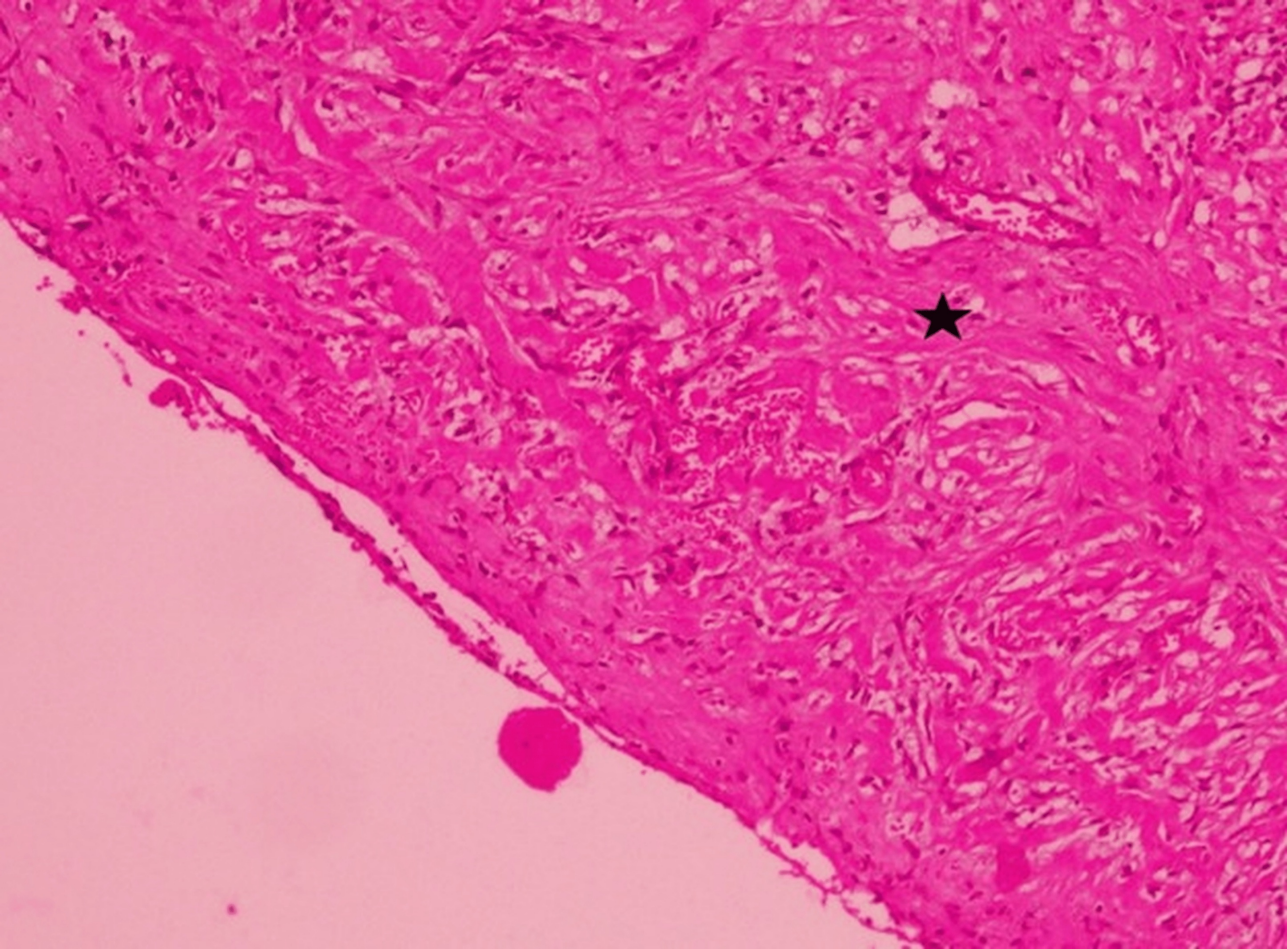
Histopathology showing fibroblastic proliferation in the cyst wall (black asterisk). H&E staining, x10.

**Figure 3:**
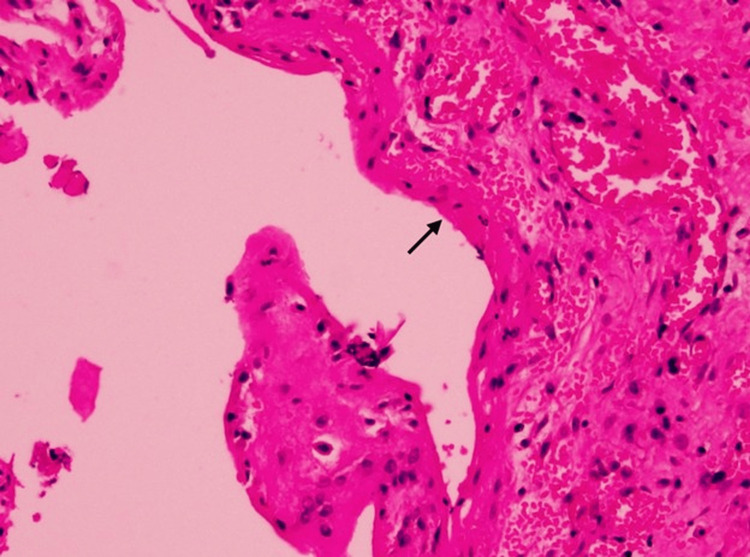
Histopathology showing flattened mesothelium lining the cyst (black arrow). Wall of cyst. H&E staining, x20.

Due to the size of the cyst, exploratory surgery was planned. An exploratory laparotomy was performed on 3 September 2025 and revealed a 6 × 4 cm cyst filled with hemorrhagic fluid and blood clots. The cyst was arising from the mesentery of the ascending colon and was strongly adherent to the omentum of the transverse colon superiorly, the appendix inferiorly, the posterolateral abdominal wall, and posteriorly to the prerenal fat. It was well encapsulated and densely adherent to surrounding structures. Deroofing and marsupialization were done, and samples were sent for biopsy. Biopsy of the excised mesenteric cyst revealed a benign, inflamed cyst wall, favoring the diagnosis of peritoneal inclusion cyst, one reactive lymph node, and no granulomatous inflammation or malignancy was seen. Three mesenteric lymph nodes showed reactive changes, and the excised omentum revealed fibroadipose tissue with fat necrosis, congestion, and dense acute on chronic inflammation with no granulomatous inflammation or malignancy seen (Figures 2, 3).

## Discussion

Mesenteric cyst was first reported in 1507 by an Italian pathologist, Antonio Benivieni, as an incidental finding during the autopsy of an eight-year-old child. Later, in 1880, Tillaux was the first to perform a successful surgery for a cystic mass in the mesentery [3,4]. With varied locations, data show that about 60% of cysts are found in the small bowel mesentery, 24% in the large bowel mesentery, and 14.5% in the retroperitoneum [5].

The exact pathophysiology of mesenteric cysts remains unclear. Obstruction due to trauma, infection, or neoplasm, or lack of communication between lymph nodes and the lymphatic or venous system, are among the most accepted theories. The cyst can be fluid-filled, chylous, or hemorrhagic, as seen in our case [5].

Although mesenteric cysts can occur in any age group, most cases have been reported in the pediatric age group of 15 years or under. The clinical presentation ranges from asymptomatic findings to symptoms of acute abdomen, as observed in our case. Symptoms usually result from compressive effects of the cyst on adjacent organs, depending on its size and location. Very few cases have led to severe complications such as intestinal obstruction or hemorrhage.

Mesenteric cysts require detailed history taking and thorough clinical examination, followed by blood tests and radiological imaging, including ultrasound and abdominal X-ray, to narrow the differential diagnoses. However, definitive diagnosis still requires histopathological confirmation. Relevant differential diagnoses include enteric duplication cyst, cystic lymphangioma, and peritoneal mesothelial or inclusion cyst.

Treatment is recommended when the cyst is symptomatic or causes complications. The treatment of choice remains surgical removal due to the low risk of recurrence, despite the availability of other modalities such as simple drainage or enucleation. Complete surgical excision has been associated with better prognosis, reducing the need for multiple follow-ups and minimizing physician and patient burden [3,6].

## Conclusions

This case highlights that mesenteric cysts, although often asymptomatic, can present with significant symptoms in infancy and mimic other causes of acute abdomen, making diagnosis challenging. With this report, we hope to contribute to the limited literature available on mesenteric cysts and to provide insight into the different symptomatic presentations that may occur. As clinical presentation can vary, further reporting and documentation are needed to facilitate earlier diagnosis and timely treatment.
